# Clinical risk in remote consultations in general practice: findings from in-COVID-19 pandemic qualitative research

**DOI:** 10.3399/BJGPO.2021.0204

**Published:** 2022-06-29

**Authors:** Rebecca Rosen, Sietse Wieringa, Trisha Greenhalgh, Claudia Leone, Sarah Rybczynska-Bunt, Gemma Hughes, Lucy Moore, Sara E Shaw, Joseph Wherton, Richard Byng

**Affiliations:** 1 Nuffield Trust, London, UK; 2 Nuffield Department of Primary Care Health Sciences, University of Oxford, Oxford, UK; 3 Community and Primary Care Research Group, University of Plymouth, Plymouth, UK

**Keywords:** remote consultations, clinical risk, general practice, family practice, telemedicine, safeguarding

## Abstract

**Background:**

The COVID-19 pandemic-related rise in remote consulting raises questions about the nature and type of risks in remote general practice.

**Aim:**

To develop an empirically based and theory-informed taxonomy of risks associated with remote consultations.

**Design & setting:**

Qualitative sub-study of data selected from the wider datasets of three large, multi-site, mixed-method studies of remote care in general practice before and during the COVID-19 pandemic in the UK.

**Method:**

Semi-structured interviews and focus groups, with a total of 176 clinicians and 43 patients. Data were analysed thematically, taking account of an existing framework of domains of clinical risk.

**Results:**

The COVID-19 pandemic brought changes to estates (for example, how waiting rooms were used), access pathways, technologies, and interpersonal interactions. Six domains of risk were evident in relation to the following: (1) practice set-up and organisation (including digital inequalities of access, technology failure, and reduced service efficiency); (2) communication and the clinical relationship (including a shift to more transactional consultations); (3) quality of clinical care (including missed diagnoses, safeguarding challenges, over-investigation, and over-treatment); (4) increased burden on the patient (for example, to self-examine and navigate between services); (5) reduced opportunities for screening and managing the social determinants of health; and (6) workforce (including increased clinician stress and fewer opportunities for learning).

**Conclusion:**

Notwithstanding potential benefits, if remote consultations are to work safely, risks must be actively mitigated by measures that include digital inclusion strategies, enhanced safety-netting, and training and support for staff.

## How this fits in

During the COVID-19 pandemic use of remote consultations expanded rapidly to reduce risk of transmission of SARS-CoV-2. As remote consultations become more established, there is a need to acknowledge and address the risks associated with them. These risks include digital exclusion, inefficiency, technology failure, and potential compromises to the quality of the consultation. To optimise use of remote consultations, stakeholders need to be involved in making decisions on the extent to which competing risks are mitigated.

## Introduction

The COVID-19 pandemic drove a sudden increase in remote consulting, with research reporting mostly telephone consulting, limited use of video, and a substantial increase in text messaging.^1^ Before the pandemic, there had been a steady increase in remote consulting (telephone, video, online, and text-based consultations) in UK general practice in response to sustained pressure from policymakers through, for example, the *General Practice Forward View*,^2^ the *Digital-first Primary Care* review,^3^ and changes to the national general practice contract. New technologies, such as AccuRx and various e-consultation services, made it easier to provide remote care.

Through successive waves of the COVID-19 pandemic, enthusiasm for remote consultation services has fluctuated. One reason for this is the changing balance between benefits and harms as the situation moved from one dominated by the risk of infection^4^ to a ‘new normal’ concerned about the impact of remote consulting on, for example, adequacy of assessment, equity, continuity of care, the therapeutic relationship,^5–7^ and overall use of NHS services. Various reports have highlighted potential problems that may arise during remote consultations,^1,8,9^ but none have yet collected and systematically analysed data from clinicians and patients about the risks they experienced while consulting remotely.

In this article, data is presented from clinicians and patients to support a taxonomy of different kinds of clinical risks associated with the ‘new normal’ of remote general practice and the article considers how these might be mitigated.

## Method

### Study design, setting, selection of data sources, and data collection

This qualitative sub-study on clinical risk in general practice drew on data selected from the wider datasets of three large, multi-site, mixed-method studies of remote care before and during the COVID-19 pandemic in the UK,^10–12^ all collected by members of the research team (summarised in Table 1 below).

**Table 1.** Data sources and methods

Title, funder, and dates	Key focus and setting	Full sample and outline methods	Subset of data used in this analysis
Remote-by-default care in the COVID-19 pandemic,UK research and innovation,June 2020–November 2021^11^	Remote assessment of unwell patients with possible COVID-19 in general practice. Four locality-level case studies in South Wales, Oxfordshire, Plymouth, and south London.	Qualitative interviews and focus groups (114 patients, 72 clinicians). Delphi study on clinical assessment (69 participants). Thirty national stakeholder interviews.	Interviews with 46 clinicians and 12 patients.
‘Near Me’ evaluation,Scottish Government,August 2019–December 2020^12^	Evaluation of Scotland’s video consultation services immediately before and during the COVID-19 pandemic, covering both primary and secondary care.What are the individual, organisational, and system-level challenges to introducing remote consultation services at pace and scale and routinising such services?	223 interviews across 17 sites with clinicians, healthcare, and third sector support workers, clinician and non-clinical managers, administrators, IT support staff, patients and their relatives, and national-level stakeholders. Ethnography across 11 sites.	Preliminary NVivo (version 12) search for the term ‘risk’ in interviews with 120 clinicians and 21 patients followed by in-depth analysis of 23 clinician and 2 patient interviews.
Video consultations,Health Foundation,June 2020–July 2021^10^	Spread and scale-up of video consultation services in primary and secondary care in England, Scotland, Wales, and Northern Ireland.What are the individual, organisational, and system-level challenges to introducing video consultation services at pace and scale and routinising such services?	National survey of 809 NHS staff. Interviews with 40 NHS staff, with 20 follow-ups across hospitals and general practice. 10 patient interviews plus 2 focus groups with 15 patients and public representatives in each.7 locality case studies, of which 3 were of video clinics in primary care. 20 policy documents reviewed.	Interviews with 10 clinicians and 10 patients.
**TOTAL**			Interviews with 176 clinicians and 43 patients
Data management for all studies	Video and telephone recordings were transcribed, deidentified, transferred to a secure server, and uploaded to NVivo software (version 12) for detailed coding. Access to recordings and transcripts was available through the secure server to members of the research team, along with the coding framework and documents explaining the codes used.		

Thematic analysis of six scoping interviews early in the Remote-by-default (RBD) study (conducted by CL, RR, and SW) identified risk as an issue for further exploration. Interview schedules for remaining RBD interviews were modified to ask clinicians about their understanding of risk, the ways in which remote consulting altered different domains of risk, and their own experiences and stories about increased risk associated with remote consulting. Patients were asked about ‘worries and concerns’; whether remote consulting felt ‘more or less safe or more or less risky’; and what they thought might reduce risk in remote consulting.

A subset of 15 interview transcripts were then coded using NVivo (version 12) by RR, SW, and CL (with three scripts coded by all three researchers) who agreed on a coding framework and standardised the meaning of codes covering the following: definition and intensity of risk; factors shaping risk; opportunities to reduce risk; and management and response to risk. After this initial subset analysis, data were shared and discussed with the wider research team, who then searched for interviews that included data related to risk from the wider RBD study (RB and SRB); the Near Me study (JW and TG); and the Health Foundation video study datasets (GH, LM, and SS). Transcripts from 176 clinicians and 43 patients (ranging in age from young adults to those aged in their 70s, and a mix of sexes and ethnicities), were reanalysed to identify data that challenged, supported, or further developed the emerging taxonomy of clinical risk. Table 1 shows a summary of aims, recruitment, and data collection for each of the primary datasets.

### Risk framework and approach to data analysis

Data were organised around a clinically focused framework developed by the Royal Australian College of General Practitioners (RACGP), which defines risk as *‘anything that threatens a practice team’s ability to achieve its clinical objectives’.*^13^ The RACGP defines the following five domains of general practice to which risks may relate: communication and the patient—doctor relationship; applied professional knowledge and skills (including all aspects of clinical performance); population and public health (including preventive care and societal influences on health); professional and ethical role (including the clinician’s duty of care, their own wellbeing, and the responsibility to keep up to date); and organisational and legal dimensions (including information technology, privacy and confidentiality, and practice management). Using these domains to guide the analysis, selected data were matched to the different risk domains. Through iterative discussion, review, and reanalysis the validity of the five domains was tested in relation to data gathered about remote consulting. An additional domain — patient role in their care — was added to the framework to accommodate a group of comments that did not fit into the RACGP domains (see column one in Table 2).

**Table 2.** Six kinds of risk associated with remote consultations

Domain	Risks identified in this study
1. **Practice set-up and organisation**Estates, care pathways (including access), technologies (including security and privacy), workforce	1A. Insufficient appointments are available 1B. Care pathways are tortuous and involve double-handling 1C. Patients are unable to access care (including various kinds of digital exclusion) 1D. Patients choose not to access care (for example, because they do not think they are a priority) 1E. Technology is inadequate or breaks down
2. **Communication and the clinical relationship**Short term: content and tone of communication within the consultation.Long term: building and maintaining a positive, trusting therapeutic relationship	2A. Information exchange is inadequate in both content and tone 2B. Consultations are overly transactional, with important concerns unsurfaced and loss of caring routines 2C. The therapeutic relationship is not established or becomes eroded
3. **Quality of clinical care**All aspects of assessment, examination, and clinical management of patients	3A. Diagnoses are missed or delayed (for example, because physical examination is limited or impossible) 3B. Safeguarding is compromised (for example, through lack of privacy or inadequate information) 3C. Patients are over-investigated or over-treated to compensate for information deficits
4. **Patient’s role in own care**Informing and supporting the patient to play an active role in own care	4A. Excessive burden is placed on the patient to make judgements, navigate care pathways, convey their symptoms, monitor their own illness, and use equipment 4B. Opportunities for patient education and information-sharing are reduced
5. **Population and public health**Preventive care, screening; societal and family aspects of health and illness	5A. Opportunities for screening and lifestyle advice are reduced 5B. Opportunities to understand and engage with the societal and family context of illness are reduced
6. **Professional development and wellbeing**Self-care; maintaining professional attitudes and commitment; lifelong learning	6A. Clinical staff become stressed, burnt out, and demotivated 6B. Opportunities for learning and development are reduced

### Management and governance

The studies were led from the University of Oxford and overseen by an external advisory group with a lay chair and patient representation.

## Results

The findings showed that, across all sites studied, the COVID-19 pandemic has led to major changes in the organisation and delivery of care (notably a redesign of estates to accommodate infection control measures and a shift to remote forms of triage and clinical interaction); the introduction of new technologies (for triage, text messaging, and video and online consultations); and physical distancing measures (leading to fewer face-to-face interactions among staff, and between staff and patients). In the latter half of the Remote by Default study, there was also a perceived increase in the number of consultations, with staff reporting being busier than before the COVID-19 pandemic. These changes, in turn, were linked to risks in the different domains of general practice.

### Risk domain 1: Practice set-up and organisation

During the first wave of the COVID-19 pandemic, infection control measures to minimise virus transmission (for example, one-way flows and consulting room closures) often reduced the number of available appointments (risk 1A in Table 2), especially for face-to-face assessment. Everyone seeking care was required to go through triage and pre-assessment, risking inefficiency owing to ‘double-touches’ with patients (risk 1B). Premises that were too small to support infection control measures comfortably had knock-on consequences for patients and staff. This was exemplified in one practice where patients had to speak through an intercom at the front door before entering the waiting room, creating a risk to confidentiality.

As the COVID-19 pandemic eased, this situation improved somewhat, but in the interviews with patients some felt they were not a priority candidate for care, and did not even try to contact their practice (risk 1D).

Those who did attempt to access care were sometimes unsuccessful (risk 1C), for a variety of reasons relating to the following: digital exclusion, including inability to use the required technology; blocked phone lines, owing to the high call volumes (and unwillingness to persist in attempts to connect); limitations on patients’ telephone data package (lack of phone minutes or bandwidth for video consultation); and not having a lifestyle that accommodates a remote callback service, as the following quote illustrates:

*'They* [patients experiencing homelessness] *don’t hang around and wait for the phone call at* [name of hostel] *... And of course you can’t reach them on a mobile because they normally don’t have one. So there was this concern that we were going to miss this group of patients, but actually it did start to work out all right. And then the patients realise they can still come and knock on the door.* […] *It helped them a bit and it wasn’t so daunting. But it was a learning curve for all of us.'* (RBD practice manager SR)

Notably, some vulnerable groups — particularly people with physical disabilities and some with mental health problems — were positive about changing to remote consulting, reducing the need to travel and wait in crowded waiting rooms. One nurse interviewee reported that she had reviewed long-term conditions in some people who had not attended for in-person reviews for many years, implying that the need to travel to and wait in the surgery had been a barrier to engagement with care.

The technologies for remote triage and consulting were more or less reliable depending on the practice’s digital maturity (risk 1E). For some, video consultations were not available at all. In others, video infrastructure was in place but if a glitch occurred, some staff struggled to cope and created workarounds, causing stress for patients and staff alike (see below, risk 6A). In practices where staff were able to switch between technologies when necessary, this could add value to consultations but swallowed up time:

*'There was a patient who phoned with abdominal pain and fever and she couldn’t speak English very well. So, we eventually got a ... we got an interpreter to do a three-way conversation who spoke to her over the phone, navigated her how to get into Near Me and then we did a video assessment of her at home. She had a ... I think one of her family members was there who was able to do some self-examination or directed examination for us to feel her stomach . And it was very time consuming, but it ended up ... we ended up admitting her as well through the ... using Near Me.'* (HFVC33 GP Scotland)

### Risk domain 2: Communication and the clinical relationship

The dataset contained many examples of incomplete, inaccurate, or misunderstood communication between patient and clinician as a result of consulting remotely (risk 2A). In the context of telephone consultations using the LanguageLine interpreting service, with no visual clues available, some patients struggled to describe their condition and GPs were sometimes uncertain about whether they had understood a patient’s problems correctly. On occasions, GPs reported long waits for a LanguageLine interpreter, leaving them feeling rushed once the consultation started.

GPs talked of the consultation becoming less rich, more transactional, and more awkward in nature, characterised by less active listening and less attention to the emotional dynamics of the interaction (risk 2B) as the following comment illustrates:

*'… And I think it’s all a wee bit false and artificial … on the video … probably a wee bit more conscious of myself and my body language and all that sort of stuff . And that there’s less … I think there’s less informal chats.'* (HFVC06 GP)

There were other accounts of the pressures faced by patients during remote consultations, which risked the quality or accuracy of information communicated to the clinician:

*‘I have a patient who is deaf, she is 30 years old. We called her husband. We have got consent that we can contact him. So once we called him up to speak about a problem, and he also has autism and he just couldn’t cope with my questioning. I was asking too many … it sent him to a little overload and he couldn’t provide the answers.'* (Practice, RBD practice nurse GI)

A related, more long-term risk is that without the depth of information and understanding gained from an initial face-to-face consultation, building and maintaining the therapeutic relationship becomes more difficult (risk 2C). While some GPs reported experiencing a good rapport with patients through remote consultations, this could be harder for patients with complex health or social situations. An initial in-person consultation was considered important to assess patients holistically, including the family and home situation, in order to establish rapport. As one GP explained:

*‘… generally if it’s complex patients who were deteriorating, I would do a telephone, I would do some investigations, bloods, and then I would do a home visit myself. And that didn’t change it, because I couldn’t … really … I did it with video a couple of times. But … I did a home visit, because I wanted to do a holistic assessment of them and examine them myself. And also just that building that trust and relationship in the first instance.'* (RBD GP KS)

### Risk domain 3: Clinical care

The majority of remote consultations in general practice occurred by phone. Some GPs reported that the time needed to explain to patients how to join a video call was not worth the additional information gained, so they abandoned this medium. Those who did use video commented that it sometimes provided clinically crucial information.

Clinicians were also concerned about missed diagnoses owing to loss of non-verbal cues, which could alert them to clinical problems (risk 3A) or safeguarding concerns (risk 3B) that went unreported by patients. To some extent these risks could be mitigated by taking a full clinical history. Several interviewees argued that the changes wrought by the COVID-19 pandemic had restored the art and the importance of history-taking, including checking for ‘red flag’ symptoms indicating serious acute problems:

*'I thought they had back pain and they’d given me a generally sensible history of back pain with … so, no leg weakness, no urinary symptoms, no bowel symptoms; I would say, "Look, you know, it sounds like you’ve got back pain; it should get better after a couple of weeks … However ..." — and this is where I do the red flag, "If this, this and this happens ... so leg numbness, bowel weakness, bladder weakness", and in the main I’d follow that up with a text message and a patient information leaflet, so that they had the information with them. We would follow that up and make sure it was all documented just to make sure*.' (HFVC19 GP Wales)

The clinical consultation is a dialogue, in which every utterance of one party is in response to, and in anticipation of, an utterance from the other party. For this reason, the asynchronous nature of online consultations, while effective for some clinical problems, posed a risk of inadequate assessment (and hence incorrect diagnosis). It was not uncommon to find that the patient’s given reason for consulting differed from their most important clinical symptoms and detailed probing — over the phone — was needed to identify their main problem:

*'*[On the text or email] *maybe a vague symptom that doesn’t really allow you to respond … and it sort of forces you to phone up and say “well, actually sorry I didn’t quite follow what you’re saying about that tummy ache”, and then you realise that actually there was a bit more going on there and you didn’t respond to that with that text about the eczema because that’s not really what they wanted to talk about, it’s that hidden agenda thing. That is no small part of what we do as GPs and it’s all a bit unspoken and difficult to quantify.'* (RBD GP CK)

The loss of precise appointment times noted above led to patients being called in workplaces and shops, on public transport or at home surrounded by other people. In some cases this created a risk that patients might withhold or distort the clinical or personal information provided (risk 2A). Several people also raised safeguarding concerns about situations where abusive partners or family members were present but out of sight during a consultation, controlling the information given to the doctor (risk 3B).

An obvious risk of a remote consultation is that elements of care, which need physical co-presence, particularly physical examination, become impossible or logistically awkward, leaving the clinician without some key information needed to reach a diagnosis. As the COVID-19 pandemic progressed, some practices reinstated face-to-face appointments and invited patients in for a physical examination if needed. Others continued with fewer or no face-to-face appointments, filling gaps in information they would have obtained from physical examination through blood tests and other investigations or through referral to a specialist. Such an approach had mixed success, as the following quote illustrates:

*'Thinking about the last two or three cancers that we’ve picked up. Quite honestly, I asked myself had I examined this patient face to face would I have picked up the cancer. And the answer is no. You know what, once* [in a different patient] *I did, yes, the patient had an enlarged liver. And that prompted me to do a two-week wait referral and … then it was an ovarian carcinoma with metastasis. But a lot of things I can think of that it is investigation that picks up so and fills in some gaps to the lack of face to face. Having said that, of course you will … you will jump into the examination if you feel that the history prompts you to do an examination then you have to do an examination*.' (RBC GP SQ)

This quote raises the possibility that invasive tests and imaging may be invoked as a substitute for a face-to-face examination (risk 3C), as another GP reflected:

*'So I will give you one example, which is, if I am managing somebody and I can’t go and see them, or if I can’t send them to hospital, I would if you would ask me, hypothetically … how would you manage? I’d say, I would love an investigation.'* (RBD GP KS)

Remote care also brought a risk of overtreatment — notably, the tendency to prescribe more antibiotics for suspected infections, partly for medicolegal reasons — and over-referral to specialists (risk 3C). One GP put it thus:

*'You were dealing with so many unknowns. You were having to deal with patients remotely. Patients that you haven’t seen face-to-face. The uncertainty, the unknown level, the stress level was quite high. And it did worry me about the medicolegal issues. The medicolegal risks ... I think that if you ask most people, they were prescribing more than they would normally do. Because you haven’t seen the patient you are going to prescribe amoxycillin antibiotics for a cough in case it’s a chest infection. It might not be a chest infection, but if it is and you don’t prescribe and they go on to have pneumonia then that’s not good.'* (RBD GP IP)

### Risk domain 4: The patient’s role in their own care

The dataset included many examples of patients who struggled to make judgements about whether or not to seek care; navigate often complex care pathways with digital entry-points; convey their symptoms in the digital environment; and monitor their own illness and use equipment such as blood pressure monitors or oximeters at home (risk 4A). While patients who were less sick and less impaired were often able to achieve these tasks, many patients were not. A patient with several long-term conditions, for example, described the stress of finding the right words to describe her symptoms, particularly if she was having a bad day:

*'If I’m non compos mentis then it’s hard to make it clear … I have foggy brain sometimes. I have fatigue most of the time. Today I’ve prepared myself* [for the research interview]*; I’ve had enough sleep; I’m articulate. If this was a situation where I need support from a GP, I may not be as articulate.'* (RBD patient FD )

Some patients reported feeling stressed when asked to examine themselves during a consultation and report back to the GP. Clinicians described having developed ways to guide patients; for example, how to look at their tonsils in the mirror, but it could turn into a prolonged and complicated process, which added to the burden of illness.

Another risk was the disruption of the care routines that involved explaining to patients what was wrong in an adaptive and supportive way (risk 4B). One GP, for example, described how they drew pictures of the bowel to explain diverticulitis to an older man with poor hearing so they could understand what was causing their symptoms. The GP missed this possibility with remote consulting.

### Risk domain 5: Population and public health

Population health interventions, such as screening and immunisations, stopped dead at the start of the COVID-19 pandemic (risk 5A). Cervical smears — which can only be conducted in person — were restored at different rates in different practices. Where screening was offered, some patients remained too scared of catching COVID-19 to attend for a preventive check.

In relation to diabetic foot checks, which would normally require in-person assessment, some practices developed methods to undertake a remote, modified check by telephone, taking a history and talking patients through a foot self-inspection and examination. Healthcare assistants and nurses were trained to conduct these assessments. Their perception was that while this did not allow for checking foot pulses, they were otherwise a reasonable alternative to an in-person examination. The finding that remote checks are perceived to be safe accords with other research, but there is as yet no evidence on the effectiveness or safety of this model.^14^

Remote consulting also created risks for maintaining the holism of general practice and the role of medical generalists in assessing and treating patients within their family and social context (risk 5B). The transactional nature of remote consulting described above, frequently negated accessing wider contextual information about patients. And while younger GP interviewees with less experience of following patients through their life course expressed less concern about this, older GPs feared losing a wider understanding of patients’ situations.

### Risk domain 6: Professional development and wellbeing

As mentioned in risk domains 1 and 4, some clinicians were stressed by the uncertainties of both the COVID-19 pandemic and the remote medium (risk 6A), describing the laborious and inefficient process of getting a full and clear verbal history from a patient remotely, and combing through notes to fill gaps in their understanding about a patient who they had not been able to see. In the quote below, a nurse describes the physical and psychological effects of many months of remote consulting:

*'I’m trying to try my best in my consultations, I’m really focusing. I’m really tired from listening on the phone, because it is exhausting. I’m really tired from these Zoom meetings, because it’s very different to have this sort of like overload of, you know, concentration and physically trying to do it, overload of information*.' (RBD practice nurse IM)

Stress and burnout have been shown to clearly carry a direct risk to clinical performance,^15^ but also increase the chance that a clinician will retire or leave the profession early, creating critical gaps in the workforce.

Even in those who did not report burnout, clinicians faced reduced opportunities for meeting colleagues face to face and seeking their advice about difficult consultations, limiting opportunities for learning from each other (risk 6B):

*'… the partners were saying, you know, “try to stay in your room at work as much as possible” … So it was, you know, I don’t want to spread anything, I don’t want to get anything. So I’d come into … and I just stay in my room … And then you feel more isolated because you’re not getting that general little chat about a case or … about how things are.'* (RBD practice nurse IM)

## Discussion

### Summary

Risks of remote consulting during the COVID-19 pandemic related to practice set-up, communication, clinical care, patients' role, population health, and professional development are summarised in Table 2. The study also identified ways in which remote consultation can reduce risk in certain groups such as engagement with long-term condition management by those who had defaulted from face-to-face check-ups.

### Strengths and limitations

The main strength of this study is that the combination of the three different primary studies provided a large and diverse dataset of interviews, focus groups, free-text responses and field notes representing a breadth of experience of general practice clinicians, patients, and support staff from across the UK. The (slightly adapted) framework from the RAGP worked well to organise the data around six domains of general practice. The main limitation is that because of the COVID-19 pandemic, the authors were unable to observe or record consultations directly, so can only report on risks as recounted by particpants.

### Comparison with existing literature

The findings present a comprehensive overview of risks, organised around the broad domains of general practice as identified by both clinicians and patients. They add to previous research and current guidance on remote consulting^8^ by combining clinician and patient views and describing risks associated with the breadth of general practice, spanning organisational, clinical, and patient and staff experiences, as well as highlighting the risk to the core values of general practice, which still needs to be addressed.

On the risk of digital exclusion, the findings of this study concur with a systematic review by Parker and others,^16^ which concluded that remote consulting might increase inequalities. The identification of risks to efficiency and a potential increase in workload associated with telephone assessment mirrors conclusions by Newbould and colleagues,^17^ while accounts of ‘double-touch’ encounters and increased demand support the findings of modelling work by Salisbury and others,^18^ which predicts an overall increase in workload associated with remote consulting.

McKinstry and colleagues^19^ concluded that remote consultations tend to be shorter and provide less rich information than face-to-face encounters. This may explain why some GPs in the present study reported difficulty building trust with patients when faced with more transactional consultations and less holistic assessments. In a similar vein, Mann and others identified various ways in which remote consulting affects the ability of GPs to deliver personalised care, arguing for, among other things, (1) training in how to build rapport and assess patients holistically (and patient capacity to partake); (2) ensuring that the design and organisation of remote services to offer continuity where needed, and (3) standardisation of care for common, simple areas of disease surveillance.^20^

In line with this literature and the present findings, Royal College of General Practitioners (RCGP) guidance on remote consulting emphasises the importance of the following: good history-taking; having a high index of suspicion for safeguarding problems; and making time to fully understand the issues in complex cases. Both the RCGP guidance and a similar resource from the General Medical Council^21^ offer detailed advice on how to choose between remote and face-to face consultations.

### Implications for practice

The emphasis in current guidance and training is on managing risk within face-to-face consultations. The risks identified in this study and others are not unique to remote consulting but, in the context of the pandemic and social distancing requirements, some may be more pronounced or occur differently; for example, risk of widening inequalities, risks to confidentiality, safeguarding issues, and missed diagnoses owing to lack of information from physical examination.

Unless the risks identified here are actively mitigated, remote care could lead to missed encounters (especially in vulnerable groups), missed diagnoses, weak safety netting, compromised safeguarding, erosion of trust, worsening inequalities, poor staff wellbeing, and overinvestigation and overtreatment.

As the COVID-19 pandemic subsides, risks relating to the organisation of physical space and physical distancing will diminish, but assessing and responding to patient abilities to consult remotely will remain critically important. These data highlight the importance of a range of clinical, organisational, technical, and educational factors to reduce risk and an urgent need to identify and address inequalities in access to general practice and wider services. This will necessitate a sustained and systematic effort to identify those who struggle to use remote services and to support them to do so in future or to use other methods of consulting.

In Table 3, a range of practical initiatives are suggested that have been inspired by interview data, workshop suggestions, and published reports, which could help to mitigate risk in the organisation and delivery of remote general practice.

**Table 3.** Mitigation actions to address risks in remote general practice consultations

**Design and delivery of services** Provide training for all staff to identify patients’ ability to engage with remote consulting and offer appointments according to these abilities Include patients as ‘co-designers’ of digital services and/or processes Maintain varied access routes into general practice, including in-person attendance to book appointments Support digital inclusion and preserve equity of access through actions such as peer-to-peer teaching provided by patient participation groups or signposting to local training in digital skills Invest in digital infrastructure to reduce the risk of failed or disrupted consultations **During consultations** Use remote consultations as one of several different modes to engage with patients in their individual contexts and swap between them when necessary and possible Pay attention to screening, preventive care, and lifestyle advice while consulting remotely Use training and guidance to build clinician skills in identifying and managing safeguarding concerns Develop robust quality and safety assurance processes for remote consulting **Supporting patients and staff** Improve communications to patients about how to access online services, including what type of consultation works best for different health problems and the principles to be applied when choosing between different types of consultation Provide training and guidance for all clinicians (see above) on how to use digital modes of access to general practice Develop the role of care navigators and social prescribers to support highly vulnerable patients and others to access services and to navigate between providers Work with external organisations to strengthen digital skills in vulnerable groups and to reduce digital exclusion **Addressing the needs of the wider population** Monitor use of other services and onward referral rates following remote consultations Ensure that population health initiatives and efforts to reduce inequalities are sustained alongside remote consulting

The authors anticipate that in some (but by no means all) cases, the benefits of remote consultations will outweigh the potential risks — but only with investment in these mitigating actions, careful design of remote services to promote inclusion, and the provision of alternative consultation modes if needed. However, as the risks are interrelated and not easily measured, it will be important to involve all stakeholders, especially patients, in decisions about how to achieve an acceptable balance of remote and face-to-face care.
